# Drug-Induced Pancreatitis Caused by Mesalazine in an Adolescent Man With Ulcerative Colitis: A Case Report

**DOI:** 10.7759/cureus.88117

**Published:** 2025-07-16

**Authors:** Yosuke Maezawa, Kazuya Nagasaki, Hiroyuki Ariga, Junya Kashimura, Hiroyuki Kobayashi

**Affiliations:** 1 Department of Internal Medicine, Mito Kyodo General Hospital, Mito, JPN; 2 Department of Gastroenterology, Mito Kyodo General Hospital, Mito, JPN

**Keywords:** acute pancreatitis, drug-induced pancreatitis, inflammatory bowel diseases, mesalazine, ulcerative colitis (uc)

## Abstract

Drugs are a rare cause of acute pancreatitis. Mesalazine, a medication used to treat inflammatory bowel disease (IBD), has been reported to cause drug-induced pancreatitis. Here, we report a case strongly suggestive of mesalazine-induced pancreatitis. A 20-year-old man presented with left upper abdominal pain after eating. He was diagnosed with acute pancreatitis based on clinical symptoms, elevated amylase levels, and findings from contrast-enhanced computed tomography. Notably, he had been diagnosed with ulcerative colitis (UC) and had started mesalazine therapy six months earlier. Mesalazine was strongly suspected as the causative agent and was subsequently discontinued. Other potential causes of pancreatitis, such as alcohol use and gallstones, were ruled out. The patient's condition steadily improved after discontinuing mesalazine, along with general supportive treatment for pancreatitis. No recurrence has been observed over several years since discontinuation. Although re-administration has not been attempted, the clinical course strongly supports mesalazine as the likely cause. When evaluating pancreatitis in patients receiving mesalazine, drug-induced pancreatitis should be considered a potential etiology.

## Introduction

The etiology of pancreatitis is multifactorial, with gallstones and alcohol consumption being the most common causes. In addition to these, rare etiologies such as inflammatory bowel disease (IBD) and drug-induced pancreatitis (DIP) have also been reported.

Pancreatitis can occur in the course of IBD, including ulcerative colitis (UC) and Crohn's disease (CD) [1]. Recent studies have identified DIP as the third most common cause of pancreatitis, following gallstones and alcohol consumption [2]. Gallstones and alcohol are responsible for approximately 38% and 32% of all cases, respectively [3,4]. The global incidence of DIP is estimated to range from five to 80 cases per 100,000 adults [5], a wide variation that may reflect regional differences. Although DIP is considered a rare cause, accounting for about 0.1-2% of acute pancreatitis cases [6], it is clinically significant. Most cases are mild to moderate in severity, but severe and potentially life-threatening cases have occasionally been reported [7].

Drugs associated with DIP are classified into four categories (Classes 1-4) based on the strength of evidence linking them to the condition [8]. Class 1 drugs have the strongest association, while Class 4 drugs have the weakest. Furthermore, Class 1 drugs are subdivided into Class 1a and 1b. Class 1a drugs are most strongly implicated in DIP, with positive rechallenge test results and no alternative causes. Class 1b drugs also show positive rechallenge results, but the presence of other potential causes cannot be excluded. Common medications in Classes 1a and 1b include mesalazine, corticosteroids, antihypertensives, and antibiotics [6,9].

Mesalazine is one of the most frequently used drugs for treating IBD and is classified as a Class 1a agent in the DIP categorization. In patients with no other identifiable risk factors, such as gallstones or alcohol use, drugs like mesalazine should be considered as possible causes of pancreatitis.

Herein, we report a case strongly suggestive of mesalazine-induced pancreatitis in the absence of other known risk factors.

## Case presentation

A 20-year-old man presented to the emergency department with a subacute onset of left upper abdominal pain that began after dinner and gradually worsened. He had a history of UC and had been receiving mesalazine therapy for six months. He had no prior history of pancreatitis, did not smoke or use other medications, and reported no alcohol consumption.

On arrival, his Glasgow Coma Scale score was E4V5M6, and his vital signs were as follows: blood pressure, 146/76 mmHg; pulse rate, 70/min; respiratory rate, 17/min; oxygen saturation, 98% (room air); and body temperature, 37.2°C. Physical examination revealed spontaneous pain in the left abdomen with tenderness across the entire abdomen, which was most pronounced in the left upper quadrant. No signs of peritoneal irritation were observed.

Laboratory tests showed a white blood cell count of 13,500/μL and a slightly elevated serum amylase level of 176 IU/L (Table 1). Renal and liver function tests and electrolyte levels were within normal ranges. No elevations in triglyceride or immunoglobulin levels, including IgG4, were noted.

**Table 1:** Comparison of laboratory data from the day of admission through day 8 of hospitalization

Laboratory test	Reference value	Day1	Day 2	Day 4	Day 8
White blood cells (/µL)	3,300-8,600	13,500	15,600	9,700	6,600
Hemoglobin( g/dL)	11.6-14.8	16.8	16.1	14.3	15.2
Platelet (/µL)	15.8-34.8×10^4^	33.5×10^4^	34.9×10^4^	28.7×10^4^	36.0×10^4^
Prothrombin time (%)	70-130	93.9	90.1		
Activated partial thromboplastin time (APTT)(sec)	24.3-36.0	28.0	28.2		
C-reactive protein (CRP) (mg/dL)	0.00-0.30	0.03	0.04	7.88	1.00
Total protein (g/dL)	6.6-8.1	7.7			
Albumin (g/dL)	4.1-5.1	4.8	4.5	3.4	3.9
Aspartate aminotransferase (AST) (IU/L)	13-30	16	15	12	29
Alanine aminotransferase (ALT) (IU/L)	7-23	12	11	7	22
Lactate dehydrogenase (LDH) (IU/L)	124-222	188	169	160	169
Alkaline phosphatase (ALP) (IU/L)	106-322	268	264	209	192
γ-Glutamyl transpeptidase (GGT) (IU/L)	9-32	17	16	11	16
Amylase (IU/L)	44-132	176	130	49	48
Total bilirubin (mg/dL)	0.4-1.5	0.8	1.1		
Blood urea nitrogen (mg/dL)	8-20	17	13	8	4
Creatinine (mg/dL)	0.46-0.79	0.78	0.73	0.79	0.83
Sodium (mEq/L)	138-145	141	140	138	139
Chloride (mEq/L)	101-108	106	106	104	102
Pottasium (mEq/L)	3.6-4.8	3.9	4.1	4.0	4.2
Calcium (mg/dL)	8.8-10.1	8.8	8.8	8.1	8.6
Triglyceride (mg/dL)	40-149	36		66	
Blood glucose level (mg/dL)	70-109	104		80	86

**Figure 1:**
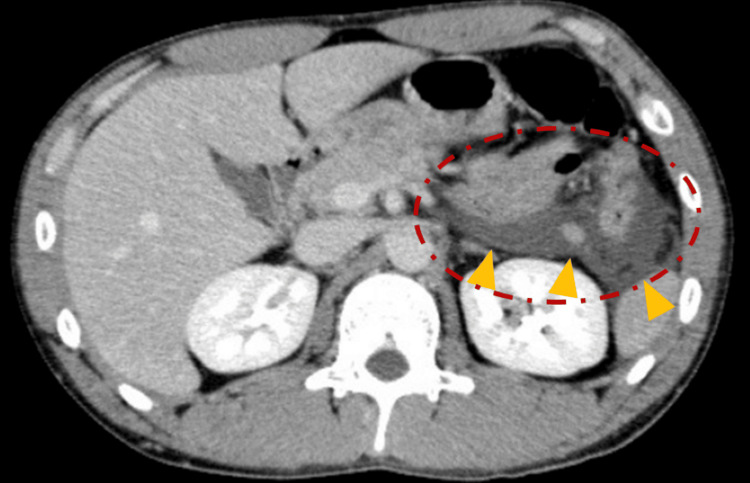
Contrast-enhanced CT revealing inflammation around the pancreas (red circle), extending into the left anterior pararenal space (yellow triangles).

**Figure 2:**
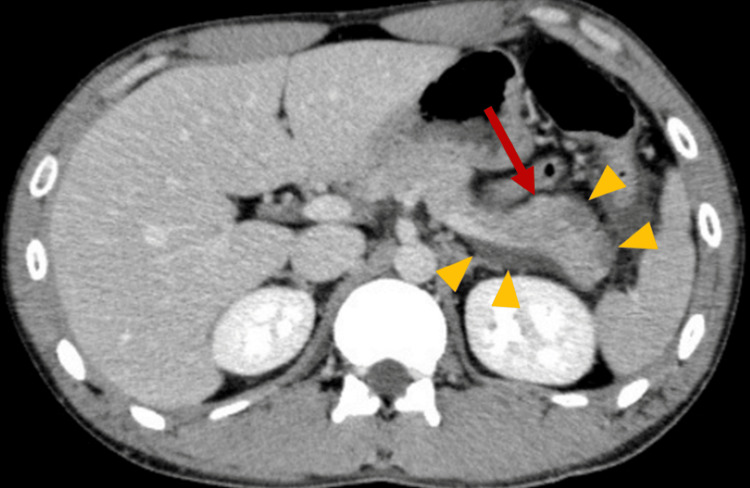
Contrast-enhanced CT showing a poorly enhanced area in the pancreatic tail, suggestive of inflammation and ischemia (red arrow), and inflammatory changes around the pancreas (yellow triangles).

Contrast-enhanced computed tomography (CT) revealed inflammation extending to the periphery of the pancreas and into the pararenal space (Figure 1), along with a poorly perfused area in the pancreatic tail (Figure 2). These findings were consistent with acute peripancreatic fluid collection (APFC) according to the revised Atlanta classification.

**Figure 3:**
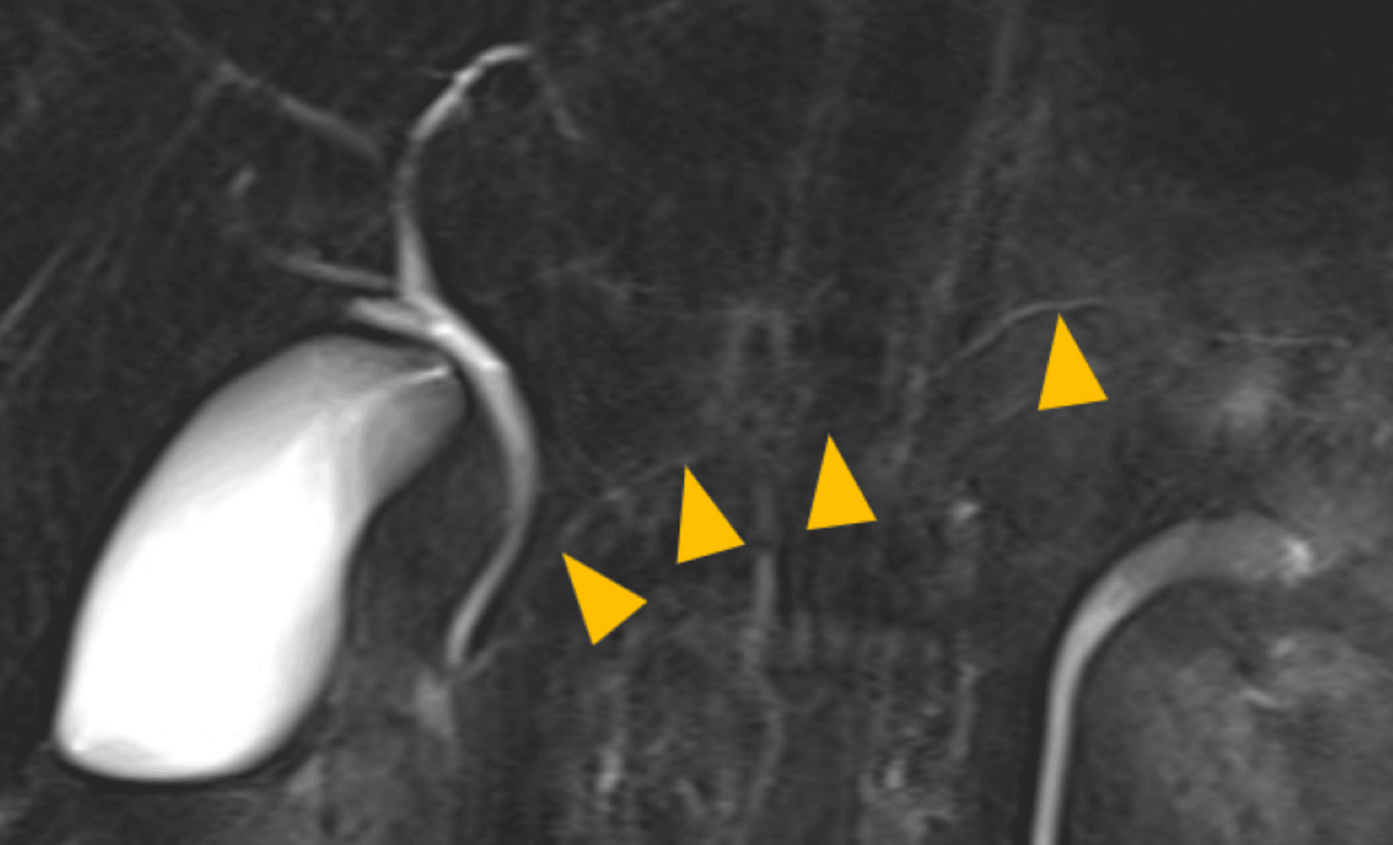
Magnetic resonance cholangiopancreatography (MRCP) demonstrating no common bile duct stones or abnormal pancreaticobiliary junction. The main pancreatic duct is indicated by yellow triangles.

Magnetic resonance cholangiopancreatography (MRCP) revealed no abnormalities, including those of the common bile duct and pancreaticobiliary malformations (Figure 3). Based on these findings, the patient was diagnosed with acute pancreatitis without prognostic factors and classified as grade 1 according to the Japan Severity Score [10].

After hospitalization, the patient was treated with fasting, fluid replacement, and protease inhibitors, following the same approach as that for acute pancreatitis caused by factors other than drugs, except for the discontinuation of mesalazine. Despite the use of pentazocine and acetaminophen, his abdominal pain persisted and required continuous intravenous fentanyl for adequate pain control. His symptoms and the amylase level improved, and he was switched to a concentrated liquid diet on day 5.

However, on day 8, the patient developed a fever of 39.3°C. His blood pressure and pulse rate remained stable, and physical examination revealed no abnormalities. Although C-reactive protein (CRP) was mildly elevated at 1.00 mg/dL on blood testing, it showed a gradual decline over time, and there was no increase in amylase levels. Contrast-enhanced chest and abdominal CT scans revealed no obvious abnormalities. In response, oral intake was discontinued, blood cultures were obtained, and empiric antibiotic therapy with piperacillin/tazobactam was initiated. Following antibiotic administration, CRP levels remained stable at approximately 1 mg/dL. The patient’s condition subsequently stabilized, and the fever resolved. Blood cultures were negative, and antibiotic therapy was completed after five days.

At the request of the patient and his family, he was transferred to a hospital closer to his home for continued care. After transfer, he resumed oral intake, remained clinically stable, and was discharged on the fourth day of hospitalization.

In this case, the patient’s symptoms developed during mesalazine administration and resolved after its discontinuation. Based on the clinical history and laboratory findings, the likelihood of alcohol-related pancreatitis or gallstone-induced pancreatitis was considered extremely low. Furthermore, there were no elevations in IgG or IgG4 levels, and imaging findings did not support a diagnosis of autoimmune pancreatitis. Therefore, we strongly suspected mesalazine-induced DIP. The Naranjo Adverse Drug Reaction Probability Scale score was 6, suggesting that mesalazine was a *probable* cause of pancreatitis in this case [11]. Given ethical concerns, a rechallenge test was not performed.

After discharge, neither mesalazine nor any other treatment for UC was reintroduced. Nevertheless, the patient's UC has remained stable, and there has been no recurrence of acute pancreatitis or development of chronic pancreatic complications.

## Discussion

The diagnosis of DIP is based on four classic criteria: (1) onset of pancreatitis during drug administration, (2) exclusion of other potential causes, (3) symptom improvement upon drug withdrawal, and (4) symptom recurrence upon re-administration of the drug [12,13]. If all four criteria are met, the association with the drug is considered *definite*. If only criteria (1) through (3) are fulfilled and a rechallenge is not performed, the association is regarded as *probable*. In our case, criteria (1) to (3) were satisfied: the onset of symptoms occurred during mesalazine therapy, no other causes of pancreatitis were identified, and the symptoms resolved after discontinuing the drug. However, a rechallenge test (criterion 4) was difficult to perform and was therefore not conducted. Accordingly, the association between mesalazine and the development of pancreatitis in this case may be considered *probable*.

DIP typically presents with clinical features of acute pancreatitis and rarely progresses to chronic pancreatitis. The prognosis is generally favorable. In many cases, an allergic reaction is implicated in its onset, and the risk is highest during the early stages of drug administration [8].

Several mechanisms have been proposed to explain the pathogenesis of DIP, including (1) pancreatic duct stenosis or obstruction, as observed with angiotensin-converting enzyme (ACE) inhibitors; (2) intracellular and extracellular enzyme activation, exemplified by valproic acid; and (3) defective intracellular transport, leading to intracellular enzyme activation caused by antiretroviral drugs, estrogen preparations, azathioprine, and other medications [14]. The exact mechanism of mesalazine-induced DIP remains unclear; however, hypersensitivity and idiosyncratic reactions are generally considered to play a role in its pathogenesis [15].

Pancreatitis caused by drug-specific toxicity is believed to occur within a short time, typically within 24 hours of administration, although it is extremely rare and has been reported for codeine and several other drugs [16]. In contrast, the pathogenesis of most DIP is thought to involve allergic reactions to medications, with pancreatitis typically developing 1-6 weeks after administration, and most often within 30 days [8]. DIP of an uncertain mechanism may be linked to the accumulation of drug metabolites and individual susceptibility. In such cases, the onset can take weeks to months or even more than a year after starting the medication [15].

For IBD, such as UC, treatments often include 5-aminosalicylic acid (5-ASA) preparations, such as mesalazine and salazosulfapyridine, as well as other immunosuppressive agents. However, these drugs are known to cause DIP, necessitating caution in their use. A Swedish case-control study found that IBD was significantly associated with an increased susceptibility to DIP, with a relative risk of 3.4 [17]. Mesalazine is generally considered to have fewer adverse effects than salazosulfapyridine. However, studies have reported that the risk of DIP in patients with rheumatoid arthritis or IBD is approximately seven-fold higher with mesalazine than with salazosulfapyridine [18]. Mesalazine is one of the most common causes of DIP in the treatment of IBD, and both the oral and enteral forms of the drug have been associated with pancreatitis. DIP typically occurs within 30 days of drug administration, most often within the first two weeks [15,19]. In a single-factor analysis, the risk of developing pancreatitis within 90 days of receiving mesalazine was reported to be 9.0 [20]. However, as seen in our case, DIP can develop more than three months after mesalazine administration; therefore, it is important to remain vigilant of the potential development of DIP.

## Conclusions

Causes of acute pancreatitis are commonly alcohol consumption and the presence of gallstones. However, it is essential to evaluate the cause based on the patient's medical history and underlying conditions to provide appropriate treatment. When common causes are ruled out, other potential causes, such as drugs, trauma, and hypertriglyceridemia, should be considered.

Several drugs used to treat IBD, including mesalazine, have been associated with DIP. If acute pancreatitis develops during treatment with these medications, DIP should be strongly suspected. Although re-administration can confirm the diagnosis, it is not always recommended due to the potential risks. When DIP is suspected, the suspected drug should be promptly discontinued. Re-challenge should only be considered when no alternative treatment options are available.

In our case, other potential causes of pancreatitis were thoroughly excluded, and the patient’s condition steadily improved following the discontinuation of mesalazine, along with standard supportive care for acute pancreatitis. No recurrence has been observed during several years of follow-up after mesalazine cessation. Although a rechallenge was not performed, the clinical course strongly supports mesalazine as the causative agent.

Notably, the onset of pancreatitis occurred approximately six months after the initiation of mesalazine therapy, considerably later than the commonly reported window of within 30 days. This case highlights that mesalazine-induced pancreatitis can occur even after prolonged exposure and underscores the importance of considering DIP as a potential diagnosis regardless of timing.

## References

[1] Choi S, Lee HJ, Seo AN (2021). Case report: development of type 1 autoimmune pancreatitis in an adolescent with ulcerative colitis mimicking pancreatic cancer. Front Pediatr.

[2] Tenner S (2014). Drug induced acute pancreatitis: does it exist?. World J Gastroenterol.

[3] Spanier BW, Dijkgraaf MG, Bruno MJ (2008). Epidemiology, aetiology and outcome of acute and chronic pancreatitis: an update. Best Pract Res Clin Gastroenterol.

[4] Ghatak R, Masso L, Kapadia D, Kulairi ZI (2017). Medication as a cause of acute pancreatitis. Am J Case Rep.

[5] Alhaddad O, Elsabaawy M, Elfauomy M (2020). Updates in drug-induced acute pancreatitis. Egypt Liver J.

[6] Jones M, Hall O, Kaye A (2015). Drug-induced acute pancreatitis: a review. Ochsner J.

[7] Wolfe D, Kanji S, Yazdi F (2020). Drug induced pancreatitis: a systematic review of case reports to determine potential drug associations. PLoS One.

[8] Badalov N, Baradarian R, Iswara K, Li J, Steinberg W, Tenner S (2007). Drug-induced acute pancreatitis: an evidence-based review. Clin Gastroenterol Hepatol.

[9] Ksiądzyna D (2011). Drug-induced acute pancreatitis related to medications commonly used in gastroenterology. Eur J Intern Med.

[10] Yokoe M, Takada T, Mayumi T (2015). Japanese guidelines for the management of acute pancreatitis: Japanese Guidelines 2015. J Hepatobiliary Pancreat Sci.

[11] Naranjo CA, Busto U, Sellers EM (1981). A method for estimating the probability of adverse drug reactions. Clin Pharmacol Ther.

[12] Kimura T, Zuidema GD, Cameron JL (1979). Steroid administration and acute pancreatitis: studies with an isolated, perfused canine pancreas. Surgery.

[13] McArthur KE (1996). Review article: drug-induced pancreatitis. Aliment Pharmacol Ther.

[14] Hung WY, Abreu Lanfranco O (2014). Contemporary review of drug-induced pancreatitis: a different perspective. World J Gastrointest Pathophysiol.

[15] Pan J, Li Z, Ye C (2025). Mesalazine-induced acute pancreatitis in inflammatory bowel disease patients: a systematic review. Ther Clin Risk Manag.

[16] Hastier P, Buckley MJ, Peten EP (2000). A new source of drug-induced acute pancreatitis: codeine. Am J Gastroenterol.

[17] Blomgren KB, Sundström A, Steineck G, Genell S, Sjöstedt S, Wiholm BE (2002). A Swedish case-control network for studies of drug-induced morbidity--acute pancreatitis. Eur J Clin Pharmacol.

[18] Ransford RA, Langman MJ (2002). Sulphasalazine and mesalazine: serious adverse reactions re-evaluated on the basis of suspected adverse reaction reports to the Committee on Safety of Medicines. Gut.

[19] Trivedi CD, Pitchumoni CS (2005). Drug-induced pancreatitis: an update. J Clin Gastroenterol.

[20] Lancashire RJ, Cheng K, Langman MJ (2003). Discrepancies between population-based data and adverse reaction reports in assessing drugs as causes of acute pancreatitis. Aliment Pharmacol Ther.

